# PD-L1 promoter methylation mediates the resistance response to anti-PD-1 therapy in NSCLC patients with EGFR-TKI resistance

**DOI:** 10.18632/oncotarget.21328

**Published:** 2017-09-27

**Authors:** Yan Zhang, Cheng Xiang, Yuling Wang, Yuanyuan Duan, Ci Liu, Yajing Zhang

**Affiliations:** ^1^ Department of Oncology, The First Hospital of Shijiazhuang City, Shijiazhuang, Hebei 050010, China; ^2^ Clinical Research Center, Shijiazhuang Fifth Hospital, Shijiazhuang, Hebei 050021, China

**Keywords:** PD-L1 promoter methylation, non-small cell lung cancer, anti-PD-1 therapy, EGFR-TKI resistance

## Abstract

The anti-PD-1/PD-L1 therapy has been demonstrated effective and safe for advanced NSCLC patients, especially for EGFR-TKIs (epidermal growth factor receptor - tyrosine kinase inhibitors) resistant NSCLC (non-small cell lung cancer) patients with EGFR mutations. However, whether the anti-PD-1/PD-L1 therapy also promotes drug resistance as EGFR-TKIs treatment remains unclear. Thus, we conducted the present study to investigate the effects of anti-PD-1 therapy on the expression of PD-L1, which is one important factor mediates the efficacy of anti-PD-1 therapy. To address the expression dynamics of PD-L1 after anti-PD-1 therapy, we first divided the patients into three groups according to the EGFR mutation status (wild type, L858R and T790M mutation). The PD-L1 was highly expressed in the NSCLC tissues than the corresponding normal tissues. After cancer recurrence, the PD-L1 was further up-regulated in patients treated with chemotherapy or EGFR-TKI therapy but decreased in the patients with anti-PD1 therapy. Promoter methylation analysis showed that the secondary NSCLC after cancer recurrence with anti-PD1 therapy had much higher promoter methylation level than the primary cancer tissue or normal tissues. In the mice model, the anti-PD-1 therapy could induce PD-L1 promoter methylation irrespective of EGFR mutation status. Combining DNA hypomethylating agent azacytidine with anti-PD-1 therapy could significantly further reduce the tumor size when comparing with the anti-PD-1 therapy alone. Our results demonstrated that the anti-PD-1 therapy might promote drug resistance through PD-L1 promoter methylation and down-regulation. And combining DNA hypomethylating agent azacytidine with anti-PD-1 therapy might be a promising approach to overcome the resistance.

## INTRODUCTION

Lung cancer is currently the most common cause of tumor related mortality in the world [1–3]. There are two main subtypes of lung cancer, small-cell lung carcinoma and non-small-cell lung carcinoma (NSCLC), accounting for 15% and 85% of all lung cancer, respectively [1–4]. Clinically, NSCLC is frequently diagnosed at advanced stages of disease [4]. Over half of patients diagnosed with lung cancer die within one year of diagnosis and the 5-year survivals are around 17.8% [1–3]. Moreover, NSCLC patients are relatively insensitive to chemo- and radio-therapy [4]. Despite advances in early detection, radical surgical resection, and multimodal therapeutic modalities over the recent decades, the long-term survival remains poor due to the high rate of recurrence and metastasis [1].

NSCLC has been delineated as a heterogeneous disease characterized by several oncogenic driver alterations, such as epidermal growth factor receptor (EGFR), anaplastic lymphoma kinase (ALK) or ROS-1 gain of function gene modifications [5, 6]. These alterations cumulatively account for approximately 20% of all cases [5, 6]. Due to increasing research on molecular tumor markers and NSCLC-driven genes, rapid development of the EGFR targeted therapy, also known as EGFR tyrosine kinase inhibitors (EGFR-TKIs), could effectively control NSCLC progression [7, 8]. Although kinase inhibitors have demonstrated significant clinical activity, their efficacy remains limited by the emergence of resistance [5, 9]. Currently, several mechanisms of EGFR-TKIs resistance have been proposed, including secondary mutations of the EGFR gene (T790M), amplification of c-Met, activation of AXL, activation of EMT, and up-regulation of IGF-1R signaling [6].

EGFR activation by EGFR mutation (such as exon-19 deletions, L858R or T790M mutation) could induce PD-L1 (programmed cell death-ligand 1) expression in advanced NSCLC [10]. Furthermore, chemotherapy and EGFR-TKI also up-regulate the expression of PD-L1 [11, 12]. Thus anti-PD-1/PD-L1 antibodies could be an optional therapy for advanced NSCLC patients, especially for EGFR-TKIs resistant NSCLC patients with EGFR mutation [9, 13–15]. PD-L1 protein expression is reported to be a potential predictor of therapy response [16, 17]. However, the response rate to anti-PD-1/PD-L1 therapy is only 15% to 45% [14]. Furthermore, some responses occur in PD-L1–negative tumors [18] while some show no responses when their tumors do not express cell surface PD-L1 [9, 14]. Thus, understanding the molecular determinants of response to anti–PD-1/PD-L1 therapy is one of the critical challenges in oncology.

Thus, we conducted the present study to investigate the expression dynamics of PD-L1 after chemotherapy, EGFR-TKI therapy or anti–PD-1 therapy in the NSCLC patients. Our results showed that both chemotherapy and EGFR-TKI therapy could up-regulate PD-L1 expression while anti–PD-1 therapy suppresses PD-L1 expression through promoter hypermethylation. Re-expressing the PD-L1 through DNA hypomethylating agent azacytidine (AZA) could sensitize the NSCLC cells to anti–PD-1 therapy *in vitro* and *in vivo* in a xenografted lung tumor model.

## RESULTS

### Reduction of PD-L1 level in NSCLC patients resistant to anti-PD-1 therapy

NSCLC patients (n=384) were divided into three groups according to the EGFR mutation status (Table 1). The group with wild type EGFR (WT group, n=214) was treated with chemotherapy (Docetaxel). The group with EGFR L858R mutation (L858R group, n=108) was treated with EGFR-TKI therapy (Gefitinib). The group with EGFR T790M mutation (T790M group, n=62) was treated with anti-PD-1 therapy (Nivolumab).

**Table 1:** Clinicopathological features

	N	EGFR status			*P* value
		Wild type (n=214, %)	L858R (n=108, %)	T790M (n=62, %)	
Age, years					0.393
<65	170	34.0	37.0	45.0	
≥65	214	66.0	63.0	55.0	
Gender					0.521
Male	180	48.4	40.7	39.1	
Female	204	51.6	59.3	60.9	
T stage					0.018^*^
T1	6	3.2	5.5	3.0	
T2	21	16.1	13.0	5.7	
T3	172	46.9	42.5	29.1	
T4	185	33.8	39.0	62.2	
N stage					0.009^*^
N0	94	67.7	48.1	40.7	
N1	158	25.7	33.3	34.7	
N2	132	6.6	18.6	24.6	
M stage					0.002^*^
M0	268	96.8	96.3	81.3	
M1	116	3.2	3.7	18.7	
AJCC stage					0.011^*^
I	72	17.7	11.0	7.2	
II	119	46.8	37.0	31.9	
III	125	32.2	48.0	42.0	
IV	68	3.3	4.0	18.9	
Differentiation					0.001^*^
High	88	66.0	50.0	32.0	
Moderate	168	27.4	36.0	41.0	
Low	128	6.6	14.0	27.0	
Vascular invasion					0.422
Yes	273	96.8	92.6	91.4	
No	111	3.2	7.4	8.6	

^*^ *P* < 0.05 indicates a significant association among the variables.

**Figure 1:**
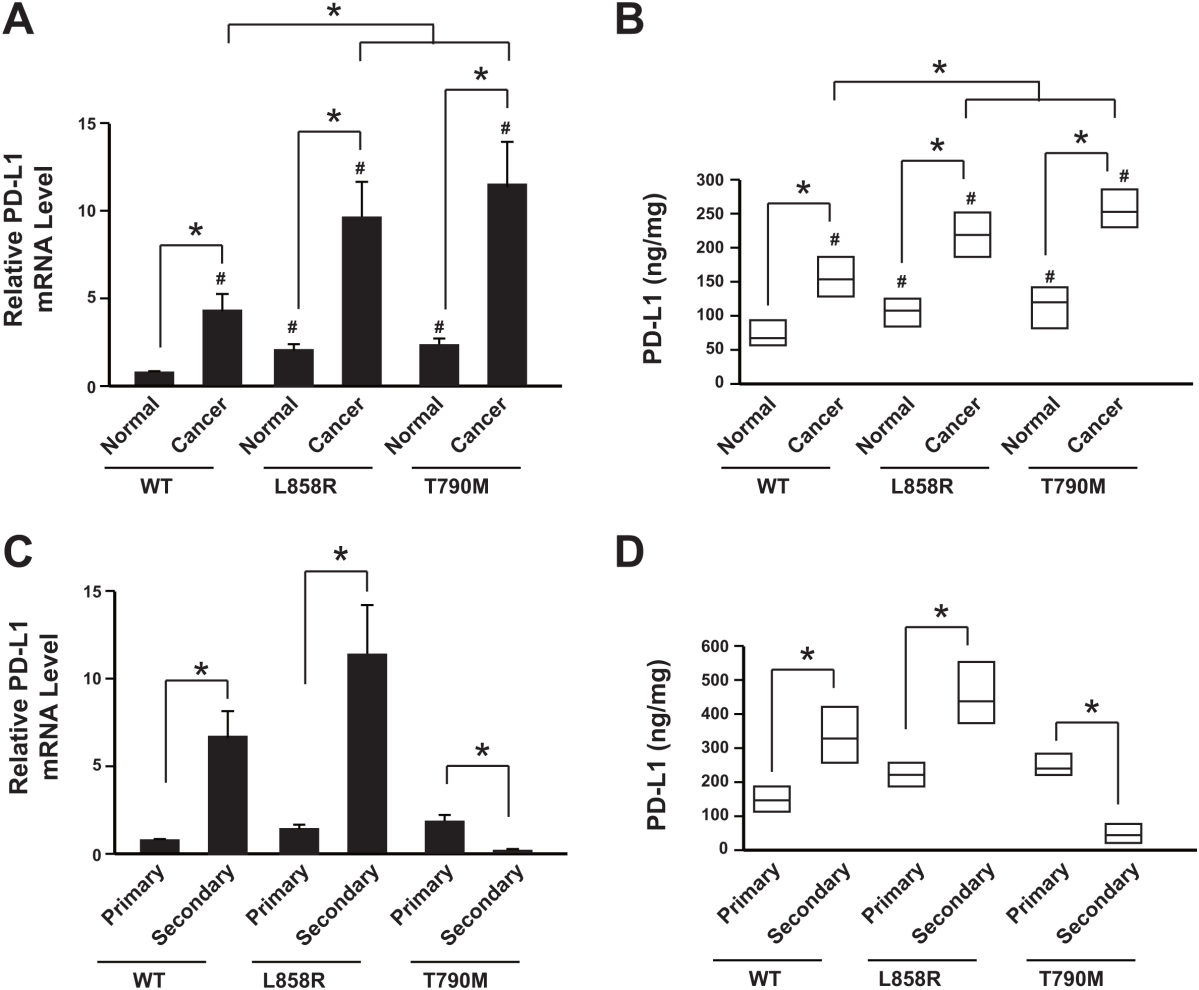
Reduction of PD-L1 level in NSCLC patients resistant to anti-PD-1 therapy **(A)** PD-L1 mRNA levels in cancer tissues and adjacent normal tissues were determined by real-time PCR. N= 214 for WT group; N=108 for L858R group; N=62 for T790M group. ^*^ *P*<0.05; ^#^ *P*<0.05 comparing with WT group normal tissue samples. **(B)** PD-L1 protein levels in cancer tissues and adjacent normal tissues were determined by ELISA. N= 214 for WT group; N=108 for L858R group; N=62 for T790M group. Result is depicted as box plots; middle line indicates median; bottom of box, 25th percentile; and top of box, 75th percentile. ^*^ *P*<0.05; ^#^ *P*<0.05 comparing with WT group normal tissue samples. **(C)** PD-L1 mRNA levels in primary cancer tissues and secondary cancer tissues after cancer recurrence were determined by real-time PCR. Data showed relative mRNA level comparing with WT group primary cancer samples. N= 214 for WT group primary cancer, N= 64 for WT group secondary cancer; N=108 for L858R group primary cancer, N= 51 for L858R group secondary cancer; N=62 for T790M group primary cancer, N= 30 for T790M group secondary cancer. ^*^ *P*<0.05. **(D)** PD-L1 protein levels in primary cancer tissues and secondary cancer tissues after cancer recurrence were determined by ELISA. N= 214 for WT group primary cancer, N= 64 for WT group secondary cancer; N=108 for L858R group primary cancer, N= 51 for L858R group secondary cancer; N=62 for T790M group primary cancer, N= 30 for T790M group secondary cancer. Result is depicted as box plots; middle line indicates median; bottom of box, 25th percentile; and top of box, 75th percentile. ^*^ *P*<0.05. WT: wild type EGFR; L858R: EGFR with L858R mutation; T790M: EGFR with T790M mutation.

We first analyzed the PD-L1 expression in 384 specimens. The data showed that both mRNA level and protein levels of PD-L1 in NSCLC tissues were higher than the corresponding normal tissues (Figure 1A, 1B). Furthermore, the PD-L1 was highly expressed in the EGFR mutation groups (both L858R and T790M groups) than the wild type group (Figure 1A, 1B).

During the follow up, recurrence was occurred (64 in WT group, 51 in L858R group and 30 in T790M group). The PD-L1 expression was further analyzed in the secondary cancer tissues. Data showed that both WT group and L858R group had increased PD-L1 level when comparing with the primary cancer tissues (Figure 1C, 1D). However, the PD-L1 level was significantly decreased in the T790M group, which was subjected to anti-PD-1 therapy, when comparing with the primary cancer tissues (Figure 1C, 1D).

Taken together, these results suggested that the PD-L1 was highly expressed in the NSCLC tissues than the corresponding normal tissues. After cancer recurrence, the PD-L1 was further up-regulated in patients with chemotherapy and EGFR-TKI therapy but decreased in the patients with anti-PD-1 therapy. This might be because of the mutation type or anti-PD-1 therapy.

### PD-L1 promoter was highly methylated in patients resistant to anti-PD-1 therapy

**Figure 2:**
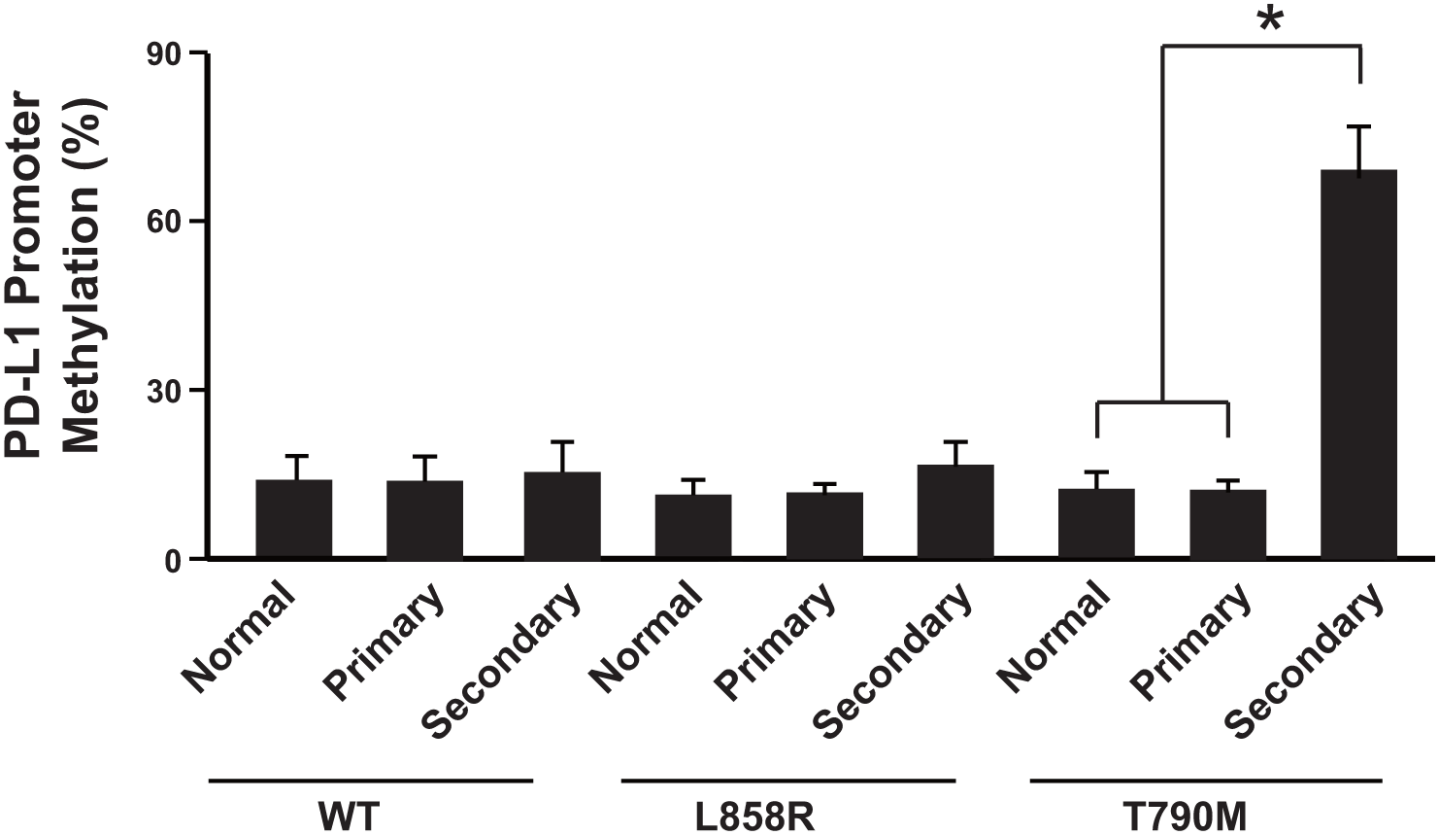
PD-L1 promoter was highly methylated in patients resistant to anti-PD-1 therapy N= 214 for WT group primary cancer and normal tissues, N= 64 for WT group secondary cancer; N=108 for L858R group primary cancer and normal tissues, N= 51 for L858R group secondary cancer; N=62 for T790M group primary cancer and normal tissues, N= 30 for T790M group secondary cancer. ^*^ *P*<0.05. WT: wild type EGFR; L858R: EGFR with L858R mutation; T790M: EGFR with T790M mutation.

To further investigate the underlying mechanism of the down-regulation of PD-L1, we studied the epigenetic modification of the PD-L1 promoter region (Supplementary Table 1). Promoter methylation analysis showed that the secondary NSCLC after cancer recurrence in T790M group had much higher promoter methylation level than the primary cancer tissue or normal tissues (Figure 2). In contrast, the WT group and L858R group did not show the same pattern (Figure 2). Thus the promoter methylation might be one of the mechanisms of PD-L1 down-regulation after anti-PD-1 therapy.

### Anti-PD-1 therapy contributes to PD-L1 promoter methylation in the mice model

**Figure 3:**
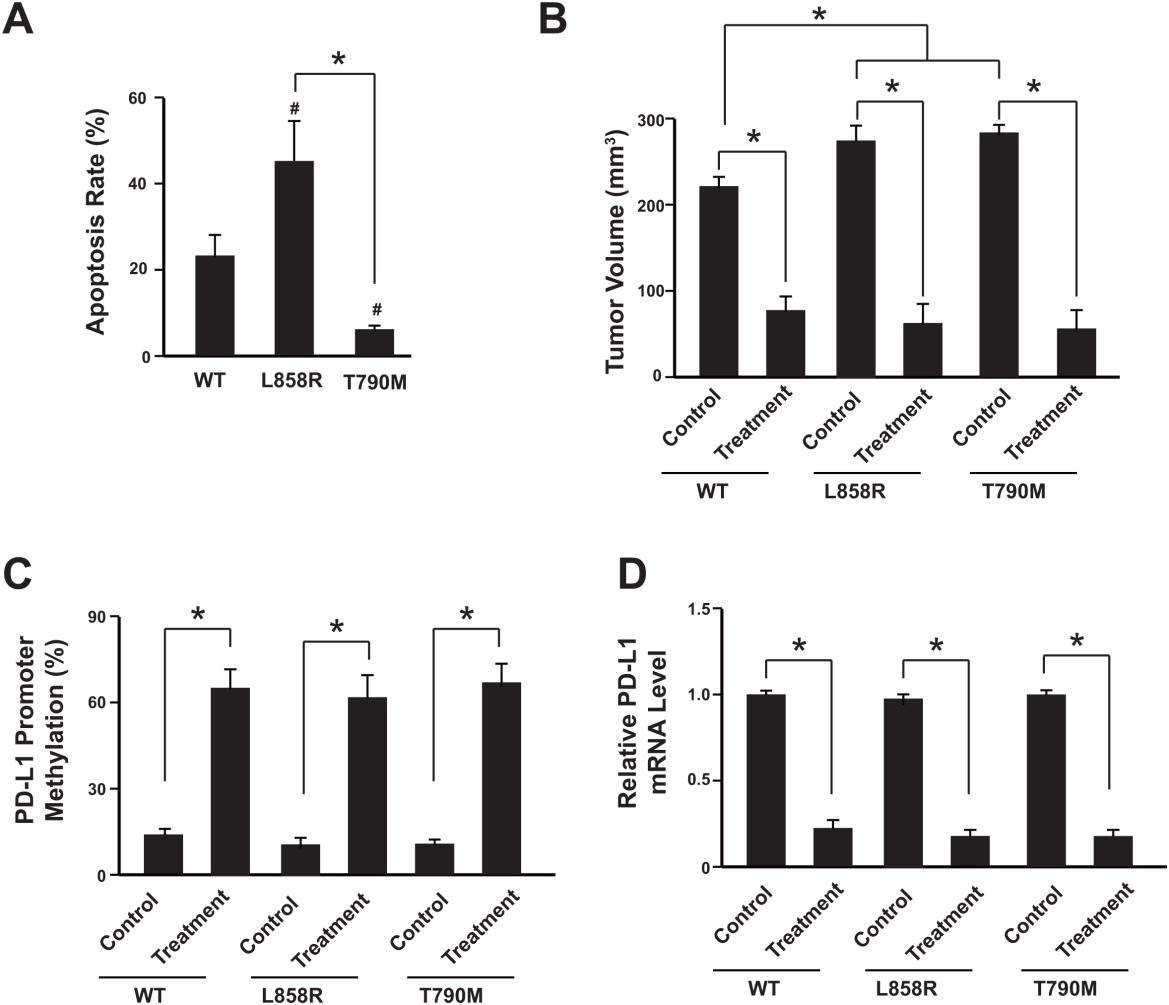
Anti-PD-1 therapy contributes to PD-L1 promoter methylation in the mice model **(A)** Analysis of NSCLC SK-MES-1 cell apoptosis following treatment of 5 μM Gefitinib for 48 hours. Cells were treated, harvested, and stained with Annexin V-FITC and 7-AAD. Annexin V-FITC-positive apoptotic cells were determined by flow cytometry. N = 3. ^*^ *P*<0.05. **(B)** The tumor volumes were analyzed 3 weeks after anti-PD-1 therapy. N = 12 for each group. ^*^ *P*<0.05. **(C)** Tumors were isolated after anti-PD-1 therapy and subjected to promoter methylation analysis. N = 12 for each group. ^*^ *P*<0.05. **(D)** PD-L1 mRNA levels were determined by real-time PCR. N = 12 for each group. ^*^ *P*<0.05. WT: SK-MES-1 with wild type EGFR; L858R: SK-MES-1 with EGFR L858R mutation created with Cas9 technology; T790M: SK-MES-1 with EGFR T790M mutation created with Cas9 technology.

To further confirm that the NSCLC cells with T790M EGFR mutation would have increased level of PD-L1 promoter methylation when subjected to anti-PD1 treatment, the NSCLC cell line SK-MES-1 with wild type of EGFR was used for establishing cell lines with EGFR mutations (L858R and T790M) via target genome editing technology. The L858R or T790M EGFR mutation was introduced into the both EGFR loci through Cas9 technology. The target mutation was validated with sequencing. And their response to EGFR-TKI (Gefitinib, 5 μM) was confirmed by cell apoptosis analysis (Figure 3A, Supplementary Figure 1).

We then used a xenograft NSCLC model to elucidate the relationship between PD-L1 promoter methylation and anti-PD1 treatment *in vivo*. SK-MES-1-WT (wild type EGFR), SK-MES-1-L858R (EGFR L858R mutation) and SK-MES-1- T790M (EGFR T790M mutation) cells were cultured, collected and injected into the mice. After each tumor reached macroscopic size, Nivolumab was administered by intravenous injection at a dose of 3 mg/kg for 3 weeks. The anti-PD-1 therapy (Nivolumab) could suppress the tumor growth significantly in all three types of cell lines (Figure 3B). After 3 weeks treatment, the tumors were isolated and the PD-L1 methylation level was measured. Interestingly, all three types of tumor harboring wild type EGFR or EGFR mutations showed increased PD-L1 promoter methylation and also decreased PD-L1 mRNA level after anti-PD-1 therapy (Figure 3C, 3D; Supplementary Figure 2).

These results suggest that anti-PD-1 therapy might induce PD-L1 promoter methylation and subsequently PD-L1 down-regulation, which would attenuate the anti-PD-1 therapy and produce resistance.

### Azacytidine sensitizes NSCLC to anti-PD-1 therapy

**Figure 4:**
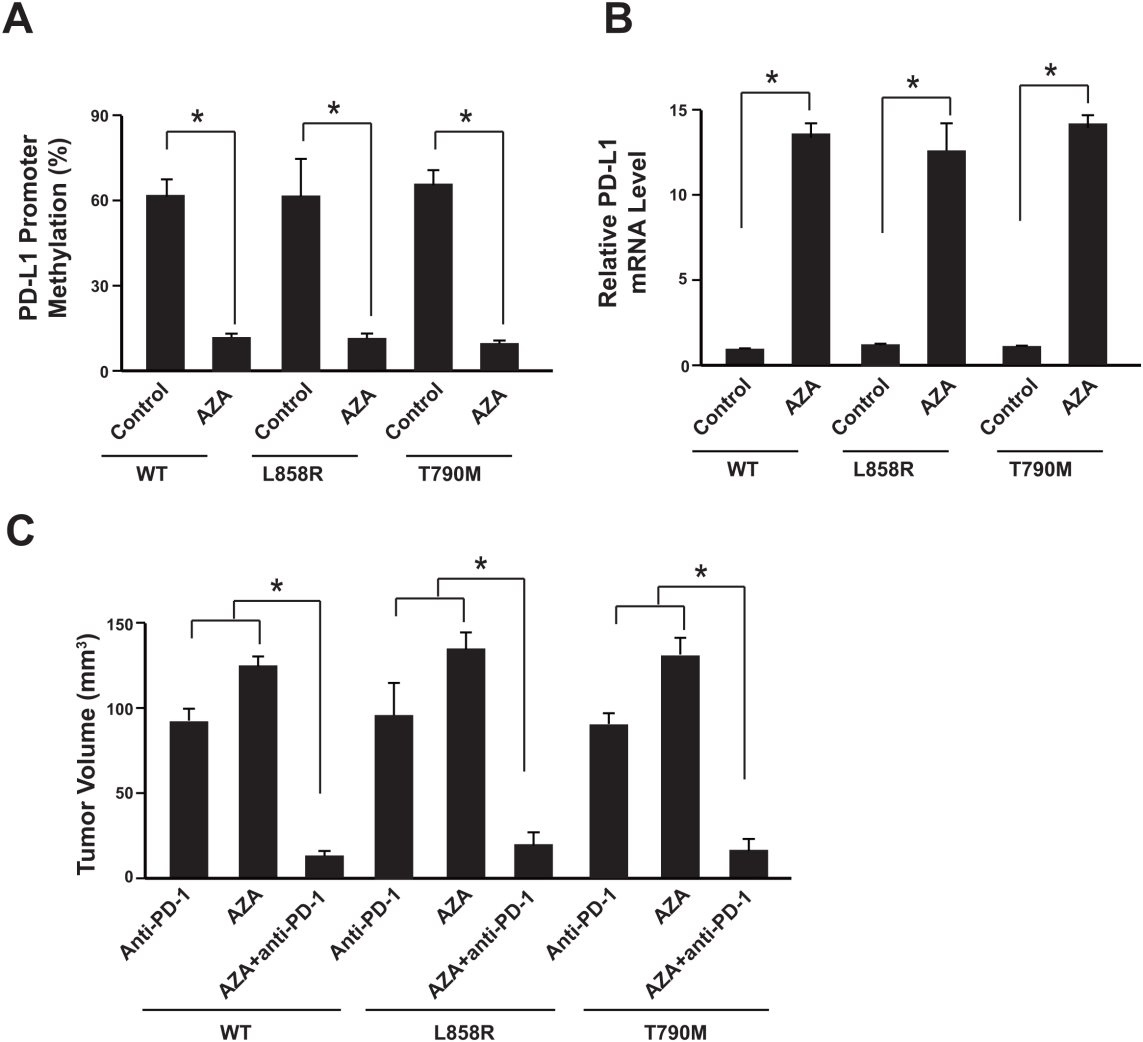
Azacytidine sensitizes NSCLC to anti-PD-1 therapy **(A)** Tumors were isolated after anti-PD-1 therapy and cells were subjected to 5 μM AZA treatment for 48 hours. Promoter methylation analysis showed methylation reduction. N=12 for each group. **(B)** PD-L1 mRNA levels were determined by real-time PCR. N = 12 for each group. ^*^ *P*<0.05. **(C)** The tumor volumes were analyzed 3 weeks after anti-PD-1, AZA or anti-PD-1 plus AZA therapy. N = 12 for each group. ^*^ *P*<0.05. WT: SK-MES-1 with wild type EGFR; L858R: SK-MES-1 with EGFR L858R mutation created with Cas9 technology; T790M: SK-MES-1 with EGFR T790M mutation created with Cas9 technology.

It has been demonstrated that the DNA hypomethylating agent azacytidine (AZA) could up-regulate PD-L1 expression [19]. Therefore, we were wondering that whether AZA could eliminate the PD-L1 promoter methylation effect of anti-PD-1 therapy and thus overcome the resistance. After 3 weeks of anti-PD-1 therapy, the tumors were isolated from the xenograft mice model and the tumor cells were subjected to AZA treatment (5 μM). Promoter methylation analysis showed that the PD-L1 methylation level was significantly reduced (Figure 4A, Supplementary Figure 3). And the PD-L1 mRNA level was increased (Figure 4B).

We next assessed the therapeutic effects of combining DNA hypomethylating agent AZA with anti-PD-1 therapy. Compared with the mice treated with Nivolumab or AZA alone, AZA plus Nivolumab could significantly further reduce the tumor size (Figure 4C).

Taken together, these results suggest that DNA hypomethylating agent AZA could eliminate the PD-L1 promoter methylation effect of anti-PD-1 therapy and sensitizes NSCLC to the anti-PD-1 therapy.

## DISCUSSION

Lung cancer is one of the most common malignancies worldwide, and it remains the leading cause of cancer related death with low early stage diagnosis rate. Due to the complexity and heterogeneity of this disease, the therapeutic strategies of patients with lung cancer differ from histological subtypes [19]. However the knowledge of biological genotypes and underlying molecular mechanisms of each histological subtype is still limited.

Several genetic alterations in NSCLC, including KRAS gene mutations, EGFR gene mutations, and EML4-ALK rearrangements, have been identified. Of these changes, EGFR gene mutations are found in approximately 10–28% of NSCLC cases [6]. Most EGFR mutations occur within the kinase domain, leading to the ligand-independent activation of EGFR signaling [20, 21]. Currently, epidermal growth factor receptor (EGFR) mutation is the most common type of gene mutations detected in Asian populations with lung cancer [22], and EGFR is identified as the therapeutic target of EGFR tyrosine kinase inhibitors (TKIs) [7, 8, 23].

Gefitinib, a common TKI, has been approved for patients harboring exon 19 deletions or exon 21 (L858R) substitution EGFR mutations [23]. This drug improves response rates, delays disease progression, and most importantly, increases overall survival compared with platinum-based combination chemotherapy. However, over the course of therapy, many patients experience resistance to TKIs [8]. Several molecular mechanisms have been described, with the EGFR T790M somatic mutation being the most frequent alteration detected in approximately half of progressing tumors [24].

Programmed cell death-1 (PD-1) pathway is a major immune checkpoint [25]. The interaction between PD-1 on T cells and PD ligand-1 (PD-L1) on tumor cells or antigen presenting cells (APCs) mutes T cell activation and T-cell-mediated tumor cell killing [26]. Antibodies blocking the PD-1 pathway, such as Nivolumab, have recently been approved by the FDA for treating several solid tumors including advanced NSCLC [9]. However, whether the anti-PD-1 therapy could promote drug resistance as EGFR-TKIs treatment remains unclear. Thus, we conducted the present study to investigate the effects of anti-PD-1 therapy on the expression of PD-L1, which is one of important factors mediates the efficacy of anti-PD-1 therapy [14, 18].

In the current study, patients were divided into three groups according to the EGFR mutation status (wild type, L858R and T790M mutation). Real-time PCR and ELISA assays showed that the PD-L1 was highly expressed in the NSCLC tissues than the corresponding normal tissues. After cancer recurrence, the PD-L1 was further up-regulated in patients treated with chemotherapy or EGFR-TKI therapy but decreased in the patients with anti-PD1 therapy. Promoter methylation analysis showed that the secondary NSCLC after cancer recurrence with anti-PD1 therapy had much higher promoter methylation level than the primary cancer tissue or normal tissues. In the mice model, the anti-PD-1 therapy could induce PD-L1 promoter methylation irrespective of EGFR mutation status. DNA hypomethylating agent azacytidine (AZA) could up-regulate PD-L1 transcripts and protein [19]. Thus combining DNA hypomethylating agent AZA with anti-PD-1 therapy could significantly reduce the tumor size when comparing with the anti-PD-1 therapy or AZA alone.

There are two potential mechanisms which might cause PD-L1 promoter hypermethylation after anti-PD-1 therapy. Firstly, the tumor cells are heterogeneous and contain both PD-L1 promoter hypermethylated cells and hypomethylated cells. The anti-PD-1 therapy could eliminate the tumor cells that overexpressing PD-L1 and having hypomethylated PD-L1 promoter. Thus the remaining cells are resistant to anti-PD-1 therapy and have hypermethylated PD-L1 promoter. On the other hand, the anti-PD-1 therapy might promote tumor cell evolution and switch off the PD-L1 expression through epigenetic modulation. So the tumor cells could escape from the anti-PD-1 therapy. However, which way contributes to the PD-L1 promoter hypermethylation after anti-PD-1 therapy remains unclear and needs further investigation.

In summary, our results showed that the anti-PD-1 therapy might promote drug resistance through PD-L1 promoter methylation and down-regulation. And combining DNA hypomethylating agent AZA with anti-PD-1 therapy might be a promising approach to overcome the resistance.

## MATERIALS AND METHODS

### Patients

A total of 384 patients were histologically verified NSCLC at the People's Hospital of Hebei Medical University between 2004 and 2014 were enrolled in this study. The median age of the patients was 55.6 years (range 29–76 years). None of them received any preoperative anticancer treatment prior to sample collection. This study was approved by the local ethics committee of People's Hospital of Hebei Medical University and written informed consent was obtained from each patient. All 384 specimens were reevaluated with respect to their histological types, differentiation status, smoking status, and tumor TNM stages. Tumor stages were determined by TNM classification according to the 2002 International Union against Cancer guidelines. The histological diagnosis and grade of differentiation of the tumors were defined by evaluation of the hematoxylin and eosinstained tissue sections, according to the World Health Organization guidelines of classification (2004). Tissues were collected within 1 h after surgery. Every patient specimen included two matched pairs, namely, NSCLC tissues and adjacent normal lung tissues (≥5 cm away from the tumor). For each specimen, half were immediately flash-frozen in liquid nitrogen and then frozen at −80 °C until DNA, RNA and protein extraction was performed, the remainder was fixed with formalin for immunohistochemistry.

Mutation of the *EGFR* gene was detected using PCR amplification and sequencing. A total of 108 patients harboring EGFR L858R mutation was administered EGFR-TKI (Gefitinib) for treatment. A total of 62 patients harboring EGFR T790M mutation was subjected to anti-PD-1 therapy (Nivolumab). And 214 patients without EGFR mutations received chemotherapy (Docetaxel).

The response to chemotherapy, EGFR-TKI or anti-PD-1 therapy was evaluated one month post-treatment by Computerized Tomographic (CT) Scanning and checked once every two months. Drug resistance and tumor recurrence was confirmed by CT. Tumor recurrence was defined as that the tumor was invisible after treatment and then became visible during the follow-up. Patients was followed-up for 36 months. The group with wild type EGFR was treated with Docetaxel (chemotherapy), 75 mg/m^2^ intravenously over 1 hour every 3 weeks and oral corticosteroids (Dexamethasone 8 mg twice a day starting 1 day prior to chemotherapy). The group with EGFR L858R mutation was treated with Gefitinib (EGFR-TKI therapy), 250 mg orally once a day. The group with EGFR T790M mutation was treated with Nivolumab (anti-PD-1 therapy), 240 mg intravenously over 60 minutes every 2 weeks. Dosage was adjusted according to the toxicity and patients responses.

### RNA extraction and real-time polymerase chain reaction (RT-PCR)

Total RNA was extracted from samples with Trizol (Invitrogen, Carlsbad, CA, USA) according to the manufacturer's instructions. Then the quantity and purity of RNA was determined by absorbance on a FilterMax F5 Multi-Mode Microplate Reader (Sunnyvale, California, USA) at 260 nm and 280 nm. Samples with ratios from 1.8 to 2.0 were accepted for next reverse transcription reaction. cDNA was prepared by using the iScript^™^ cDNA Synthesis kit (Bio-Rad, USA). β-actin was used as internal control. RT-PCR amplification reaction was prepared with the SYBR Green PCR kit (Bio-rad, USA) and performed using the 7500 fast Real-Time PCR system (Applied Biosystems, USA). PCR products were verified by melting curve analysis. Relative mRNA levels of target genes were calculated by the 2^-ΔΔct^ method.

### Enzyme-linked immunoassay (ELISA)

The protein level of PD-L1 was detected in tumor homogenate using PD-L1 ELISA Kit (R&D systems) according to the manufacturer's instructions. All samples were assayed in triplicate.

### Promoter methylation analysis

For methylation analysis, samples were processed according to the instruction of InnuCONVERT Bisulfite All-In-One Kit (Analytik Jena). For assay validation, a dilution series of bisulfite-converted, unmethylated sperm DNA (NW Andrology& Cryobank Inc., Spokane, WA, USA) and artificially methylated DNA (CpGenome^™^ Universal Methylated DNA; Merck Millipore, Darmstadt, Germany) were used. DNA concentration was quantified by UV spectrophotometry using a Nanodrop ND-1000 spectralphotometer (Nanodrop Technologies, Wilmington, DE, USA). The promoter region of PD-L1 was PCR amplified, T-A cloned and sequenced. Methylation analysis was performed by calculating the ratio of methylated CpG sites to total CpG sites.

### Cell culture

The SK-MES-1 human NSCLC cell line was obtained from the American Type Culture Collection (ATCC; Rockville, MD, USA) and cultured in DMEM (GIBCO, Shanghai, China) supplemented with 10 % FBS.

### Genome editing with Cas9

EGFR target mutation was conducted via Cas9 system [27]. Guide RNA was designed with ZiFiT Targeter (http://zifit.partners.org/ZiFiT/) [28]. The target site for L858R is 5′-CAAGATCACAGATTTTGGGCTGG-3′ and for T790M is 5′-ATCACGCAGCTCATGCCCTTCGG-3′. Targeting vector with 1kb homologous arm and puromycin resistance gene was co-transfected with Cas9 plasmids. After target mutation, the puromycin gene was deleted with LoxP-Cre system. The mutation was confirmed by sequencing.

### Animal study

Female 6–8 weeks old BALB/c nu/nu mice (Charles River Laboratories, Beijing, China) were housed in specific pathogen-free conditions. The study was approved by the Research Ethics Committee of People's Hospital of Hebei Medical University. For evaluation of the tumor growth *in vivo*, 1 × 10^7^ cells were suspended in 200 μl PBS and injected subcutaneously into the flank region of nude mice. Tumor growth was monitored every 2 days and tumors were measured with fine digital calipers and tumor volume was calculated by the following formula: tumor volume =0.5 × width^2^ × length. After tumor volume reached to around 60 mm^3^, the mice were subjected to anti-PD-1 therapy. One day before the treatment, 1 × 10^7^ human PBMC (peripheral blood monocyte cells) were transplanted into the mice via tail-vein. Nivolumab was administered by intravenous injection at a dose of 3 mg/kg. Azacitidine (AZA) was administered by intravenous injection at a dose of 3 mg/kg. After 3 weeks, the mice were sacrificed, the tumors were collected, and the tumor volumes were measured.

For tumor cell isolation and culturing, the freshly isolated tumors were washed with cold PBS supplemented antibiotics for three times. Then the tumors were minced into small pieces and incubated with 0.05% Trypsin-EDTA for 5-10 minutes with agitation. Single cells were collected and neutralized with culture medium supplemented with 10% FBS. Cells were plated into culture dish after centrifugation and subjected to drug treatment after 48 hours.

### Statistical analysis

Data were expressed as mean±SE and analyzed by Graphpad Prism V.5.00 software (GraphPad Software, San Diego CA, USA). Comparisons between groups were made using nonparametric Mann-Whitney U-test. *p* values under 0.05 were considered statistically significant.

## SUPPLEMENTARY MATERIALS FIGURES AND TABLE


