# Supplementary material for: PD-L1 promoter methylation mediates the resistance response to anti-PD-1 therapy in NSCLC patients with EGFR-TKI resistance
Source: Oncotarget. 2017 Sep 27;8(60):101535–44. doi: 10.18632/oncotarget.21328 (PMC5731894; doi:10.18632/oncotarget.21328)
Supplement: Supplementary file 1 [file oncotarget-08-101535-s001.pdf]

PD-L1 promoter methylation mediates the resistance response to anti-PD-1 therapy in NSCLC patients with EGFR-TKI resistance

SUPPLEMENTARY MATERIALS

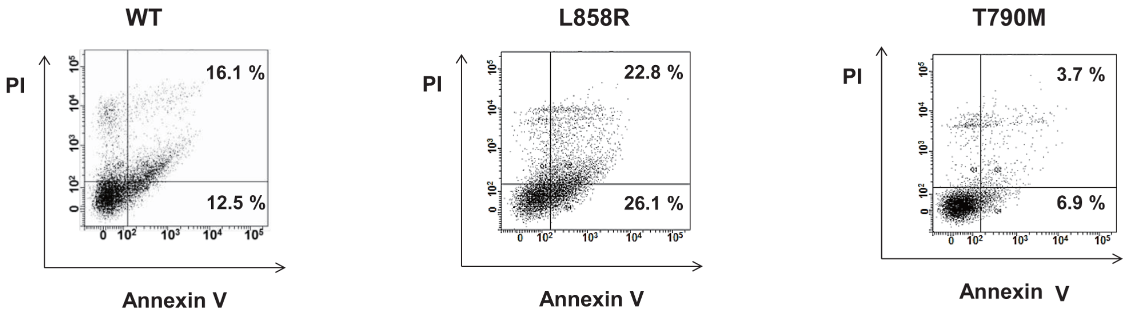

Supplementary Figure 1: Cell apoptosis analysis of NSCLC cells with EGFR mutations treated with Gefitinib. WT: wild type EGFR; L858R: EGFR with L858R mutation; T790M: EGFR with T790M mutation.

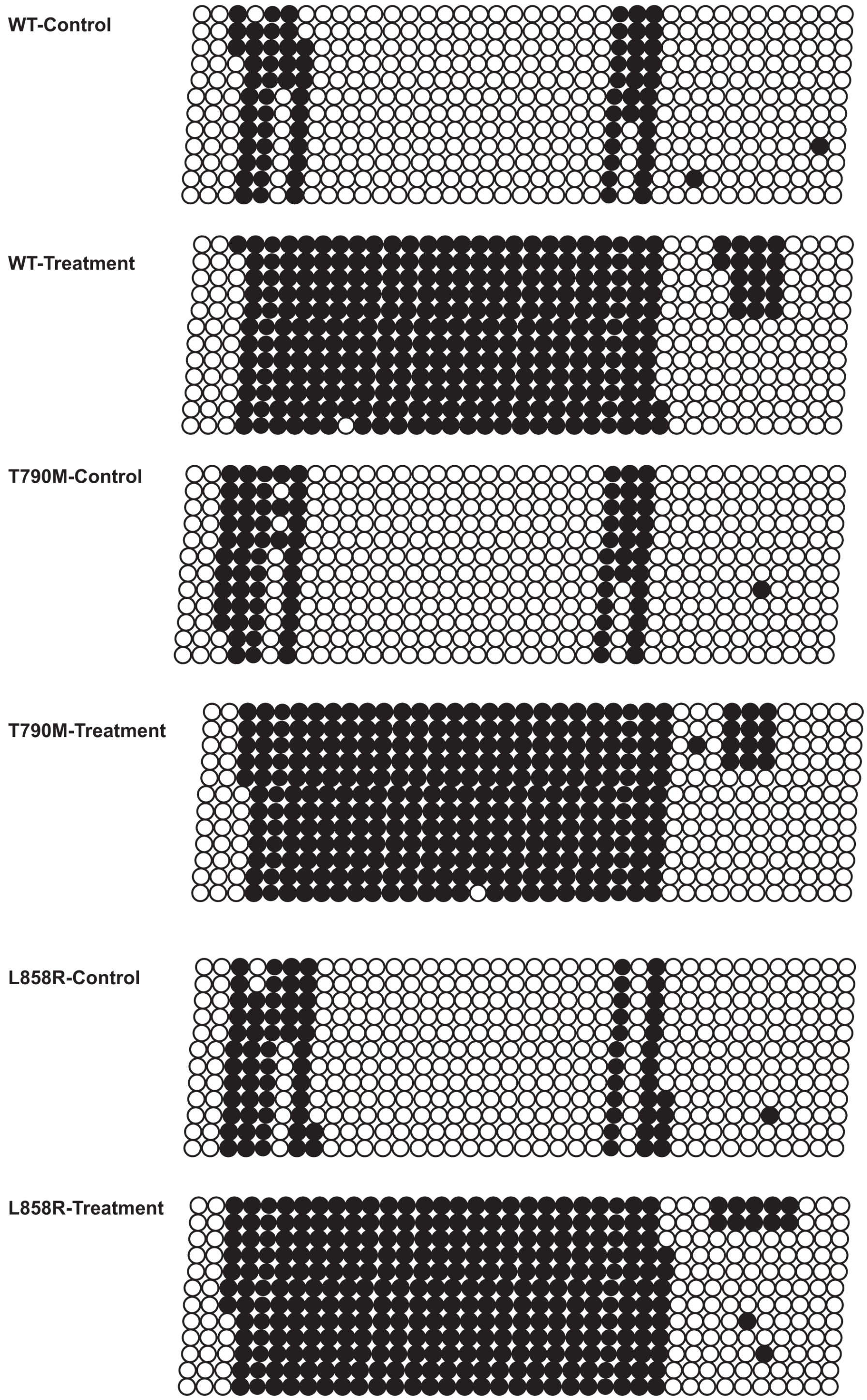

Supplementary Figure 2: Anti-PD-1 therapy contributes to PD-L1 promoter methylation in the mice model. WT: wild type EGFR; L858R: EGFR with L858R mutation; T790M: EGFR with T790M mutation.

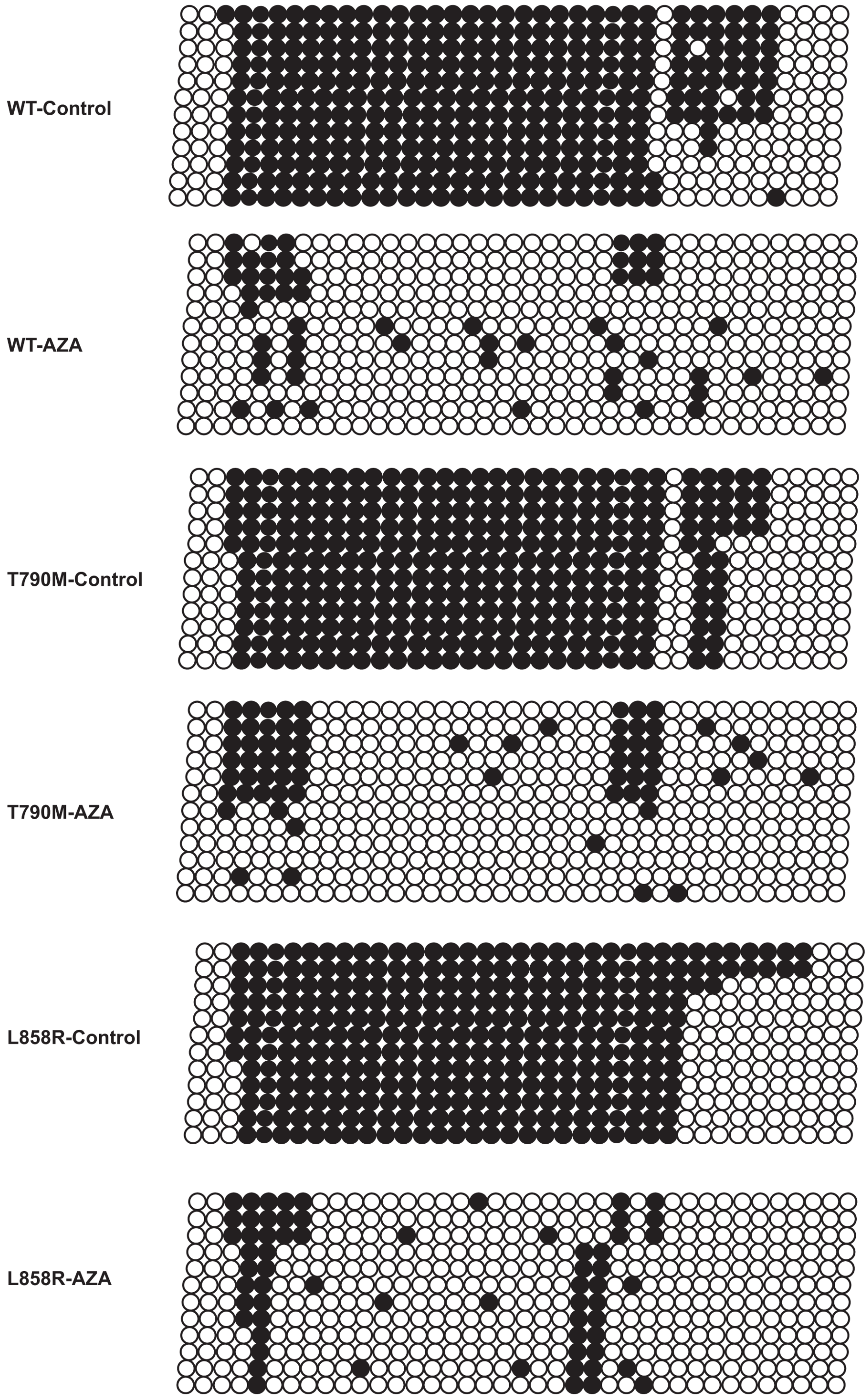

Supplementary Figure 3: Azacytidine decreases PD-L1 promoter methylation. WT: wild type EGFR; L858R: EGFR with L858R mutation; T790M: EGFR with T790M mutation.

Supplementary Table 1: Human PD-L1 promoter for methylation analysis

>Human PD-L1 Promoter for methylation analyisisAACTCTTCCCGGTGAAAATCTCATTTACAAGAAAACTGGACT
GACATGTTTCACTTTCTGTTTCATTTCTATACACAGCTTT*ATTCCTAGGACACCAACACTAGATACCTAAACTGA*AA
GCTTCCGCCGATTTCACCGAAGGTCAGGAAAGTCCAACGCCCGGCAAACTGGATTTGCTGCCTTGGGCAGA
GGTGGGCGGGACCCCGCCTCCGGGCCTGGCGCAACGCTGAGCAGCTGGCGCGTCCCGCGCGGCCCCAGTTC
TGCGCAGCTTCCCGAGGCTCCGCACCAGCCGCGCTTCTGTCCGCCTGCAGGTAGGGAGCGTTGTTCCTCCGC
GGGTGCCCACGGCCCAGTATCTCTGGCTAGCTCGCTGGGCACTTTAGGACGGA*GGGTCTCTACACCCTTTCTTT*
*GGGATGGAGAGAGG*AGAAGGGAAAGGGAACGCGATGGTCTAGGGGGCAGTAGAGCCAATTACCTGTTGGGG
TTAATAAGAACAGGCAATGCATCTGGCCTTCCTCCAGGCGCGATTCAGTTTTGCTCTAAAAATAATTTATACCT
CTAAAAATAAATAAGATAGGTAGTATAGGATAGGTAGTCATTCTTATGCGACTGTGTGTTCAGAATATAGCTCT
GATGCTAGGCTGGAGGTCTGGACACGGGTCCAAGTCCACCGCCAGCTGCTTGCTAGTAACATGACTTGTGTA
AGTTATCCCAGCTGCAGCATCTAAGTAAGTCTCTTCCTGCGCTAAGCAGGTCCAGGATCCCTGAACGGAATTT
ATTTGCTCTGTC

Letters in red color indicate the GC sites; letters in italic and underlined indicate the primer sites.
